# Association between hypomagnesemia and severity of primary hyperparathyroidism: a retrospective study

**DOI:** 10.1186/s12902-021-00838-y

**Published:** 2021-08-20

**Authors:** Ding Na, Guo Tao, Liu Shu-Ying, Wang Qin-Yi, Qu Xiao-Li, Li Yong-Fang, Ou Yang-Na, Sheng Zhi-Feng, Yang Yan-Yi

**Affiliations:** 1 National Clinical Research Center for Metabolic Diseases, Hunan Provincial Key Laboratory of Metabolic Bone Diseases, Department of Metabolism and Endocrinology and Health Management Center, The Second Xiangya Hospital of Central South University, Changsha, Hunan China; 2 Department of Surgery, the First Affiliated Hospital of Hunan Normal University, Changsha, 410005 Hunan China; 3 Hospital Infection Control Center, the Second Xiangya Hospital of Central South University, 139 Middle Renmin Road, Changsha, 410011 Hunan China; 4 Health Management Center, the Second Xiangya Hospital of Central South University, 139 Middle Renmin Road, Changsha, 410011 Hunan China

**Keywords:** Primary hyperparathyroidism, Hypomagnesemia, Severity, Biochemical, Clinical

## Abstract

**Background:**

The occurrence of hypomagnesemia in patients with primary hyperparathyroidism (PHPT) has been noted previously; however, the association of hypomagnesemia and severity of primary hyperparathyroidism remains unknown. The present study aimed to evaluate the association of hypomagnesemia with biochemical and clinical manifestations in patients with PHPT.

**Methods:**

This was a retrospective study conducted at a tertiary hospital. We obtained data from 307 patients with PHPT from January 2010 through August 2020. Data on demographics, history, laboratory findings, bone densitometry findings, and clinical presentation and complications were collected and were compared in normal magnesium group vs hypomagnesemia group.

**Results:**

Among the 307 patients with PHPT included in our study, 77 patients (33/102 [32.4%] males and 44/205 [21.5%] females) had hypomagnesemia. Mean hemoglobin levels in the hypomagnesemia group were significantly lower than those in the normal magnesium group in both males and females. In contrast, patients with hypomagnesemia had a higher mean serum calcium and parathyroid hormone than individuals with normal magnesium. The typical symptoms of PHPT, such as nephrolithiasis, bone pain/fractures, polyuria, or polydipsia, were more common in the hypomagnesemia group. In addition, patients with hypomagnesemia had a higher prevalence of osteoporosis, anemia, and hypercalcemic crisis. Even after adjusting for potential confounders, including age, sex, body mass index, estimated glomerular filtration rate, and parathyroid hormone levels, these associations remained essentially unchanged.

**Conclusion:**

Biochemical and clinical evidence indicates that patients with PHPT with hypomagnesemia have more severe hyperparathyroidism than those without hypomagnesemia. In addition, PHPT patients with hypomagnesemia had a higher prevalence of osteoporosis, anemia, and hypercalcemic crisis.

## Introduction

Primary hyperparathyroidism (PHPT) is a generalized endocrine disorder characterized by hypercalcemia and high or inappropriately normal concentrations of parathyroid hormone (PTH) [1–3]. Patients with PHPT may experience an array of symptoms, including nephrolithiasis, bone pain/fractures, gastrointestinal symptoms, thirst, and polyuria [4]. Parathyroid hormone activates the parathyroid hormone receptor, increasing bone and distal tubular calcium resorption [4]. Parathyroid hormone also plays a role in vitamin D metabolism; it activates vitamin D 1-alpha hydroxylase, which increases the renal synthesis of 1,25-dihydroxyvitamin D to enhance dietary calcium absorption [4]. Levels of PTH may be regulated by serum magnesium [5–8]. Hypermagnesemia may cause hypocalcemia, through activation of calcium-sensing receptors in the parathyroid glands, thereby suppressing PTH secretion [5–8]. Magnesium deficiency may lead to the production of defective cyclic adenosine monophosphate in the parathyroid glands and in PTH target organs. This may be the principal mechanism underlying both impaired PTH secretion and end-organ resistance to PTH, which together contribute to the development of hypocalcemia [9–11].

Magnesium is the second most common intracellular cation [12]. Its abundance facilitates its multiple functions in common, essential intracellular processes. It is a co-factor in multiple enzymatic reactions, including those related to energy metabolism and DNA and protein synthesis, and it participates in the regulation of ion channels [13]. Magnesium is essential for the regulation of muscular contraction, blood pressure, insulin metabolism, cardiac excitability, vasomotor tone, nerve transmission, and neuromuscular conduction [14]. Although PTH stimulates an increase in tubular magnesium reabsorption in patients with PHPT, the direct effect of hypercalcemia on tubular magnesium reabsorption is the opposite. As a result, the serum magnesium levels in patients with PHPT are usually normal or only slightly reduced [15]. However, the occurrence of hypomagnesemia in PHPT has been noted previously [16–18], and patients with more severe PHPT, manifested by high serum calcium, tend to have low serum magnesium [18]. These findings suggest that hypomagnesemia has a potential in predicting the severity of hypercalcemia in patients with PHPT.

To the end, the occurrence of hypomagnesemia in patients with PHPT has been noted previously; however, the association of hypomagnesemia and severity of primary hyperparathyroidism remains unknown. In the present study, using a retrospective review of 307 consecutive PHPT patients who were continuously hospitalized, we investigated the association of hypomagnesemia with biochemical and clinical manifestations in patients with PHPT.

## Materials and methods

### Study design and patients

We performed a retrospective review of 307 hospitalized patients with PHPT who were continuously admitted to the Second Xiangya Hospital of Central South University, which is a tertiary hospital in Changsha, Hunan Province, Central South of China, from January 2010 to August 2020. The data collection was performed by trained endocrinologists. Patients diagnosed with secondary hyperparathyroidism, tertiary hyperparathyroidism, familial hypocalciuric hypercalcemia (FHH) were excluded from the study. Hypomagnesemia was defined as a serum magnesium level < 0.75 mmol/L [19]. Osteoporosis was defined, according to the World Health Organization criteria, as a bone mineral density (BMD) T score of − 2.5 standard deviations (SD) below that of a healthy young individual of the same sex; BMD is most commonly measured using dual energy X-ray absorptiometry [20]. Anemia was defined as hemoglobin < 130 g/L in males and < 120 g/L in females [21]. Hypercalcemic crisis was defined as a serum calcium level > 3.5 mmol/L [22].

This study protocol was approved by the Ethics Committee of the Second Xiangya Hospital of Central South University. All study methods were carried out in accordance with relevant guidelines and regulations. All the patients provided informed consent for participating in this study.

### Medical history collection and anthropometric information

Medical records were reviewed for age, sex, and symptoms of PHPT, including nephrolithiasis, fatigue, polydipsia, gastrointestinal symptoms, bone pain, and a history of osteoporosis and fractures. Patient height was measured to the nearest 0.1 cm, and weight was recorded to the nearest 0.1 kg, with the participant wearing light clothing. Body mass index (BMI) was calculated as weight in kg divided by height in m^2^.

### Biochemical measurements

Serum levels of calcium, phosphorus, magnesium, PTH, 25-hydroxyvitamin D (25[OH]D), albumin, and creatinine were recorded. Blood samples were collected from patients fasted overnight. Hemoglobin (Hb) levels were measured using an automated hematology analyzer ADVIA 2120 (Siemens Healthcare Diagnostics, Germany). Serum albumin, calcium, phosphorus, and magnesium, and albumin were determined using an automatic biochemical analyzer (Abbott Laboratories, North Chicago, IL, USA). Albumin-corrected serum calcium was calculated using the following formula: corrected calcium (mmol/L) = serum calcium (mmol/L) + 0.02 × (40 − serum albumin [g/L]) [23]. Serum creatinine levels were measured were measured by an enzymatic assay (Sarcosine oxidase method, Kanto Chemical, Tokyo, Japan) with a Roche Modular P800 automatic analyzer (Roche Diagnostics, Mannheim, Germany), and the estimated glomerular filtration rate (eGFR) was estimated using the CKD-EPI 2009 equations [24]. Serum PTH was measured using the automated chemiluminescence immunoassay (Siemens Healthcare Diagnostics, Erlangen, Germany). Serum 25(OH) D was measured using an enzyme-linked immunosorbent assay (Immunodiagnostic Systems Limited, Boldon, UK). Urinary calcium and magnesium were measured by a colorimetric assay (Cobas System Roche, Pleasanton, CA, USA). All inter- and intra-assay coefficients of variation were less than 10% [25].

### Bone mineral density measurement

Lumbar spine, femoral neck, and total hip BMD was measured using dual-energy X-ray absorptiometry with the GE Lunar Prodigy Advance (CV ≤ 1%; T score and Z score) in patients.

### Statistical analysis

Data that were normally distributed were expressed as mean ± SD, whereas data that did not follow a normal distribution were expressed as median (range). Values between groups were compared using Student t-test and Wilcoxon rank sum test for normally and non-normally distributed data, respectively. Spearman correlation coefficients were used to assess the association between serum magnesium and other variables. Linear regression was used to find relations between serum magnesium and other variables. Unadjusted and adjusted odds ratios were analyzed with logistic regression models. Statistical significance was set at *P* < 0.05 (two-sided). All statistical analyses were performed using the SPSS software application (version 22.0: SPSS, Chicago, IL, USA).

## Results

**Fig. 1:**
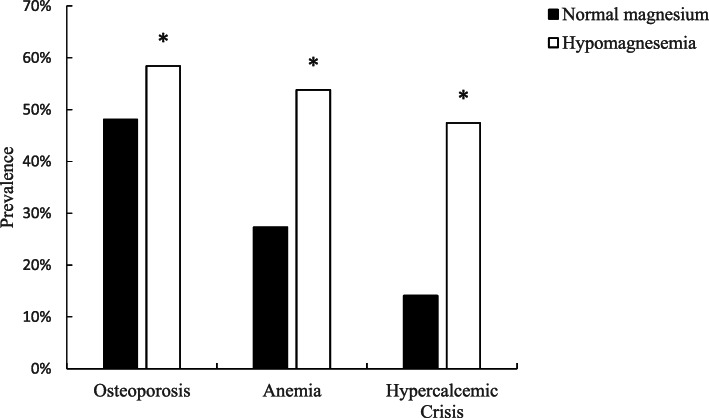
Prevalence of complications of PHPT patients. *vs normal magnesium group, *P<0.05*

**Fig. 2:**
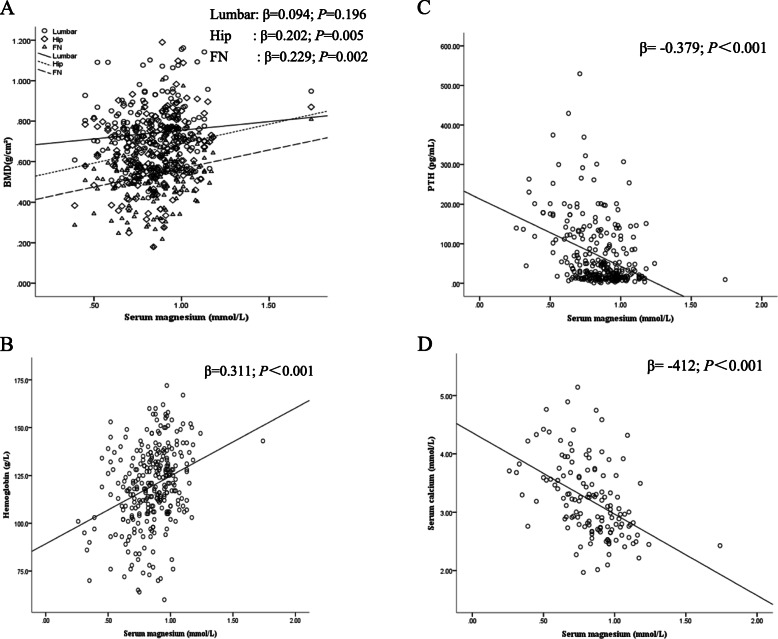
Relationship between serum magnesium and various indicators in patients with PHPT

Among the 307 patients with PHPT in the present study population, 102 were males and 205 were females. The mean age of the study population was 52.2 ± 14.6 years. Seventy-seven patients (33 [32.4%] males and 44 [21.5%] females) had hypomagnesemia, accounting for 25.1% of the total population (Table 1). There was no difference in the duration of disease and serum phosphorus and 25(OH) D levels between the two groups. The average hemoglobin in the hypomagnesemia group was significantly lower than that in the normal magnesium group in both males and females; patients with hypomagnesemia also had a higher prevalence of anemia (53.8% versus 27.3%, *P* < 0.001; Fig. 1). The hemoglobin levels were positively correlated with serum magnesium levels (*P* < 0.05; Fig. 2). The average serum calcium in the hypomagnesemia group was significantly higher than that in the normal magnesium group in both males and females; patients with hypomagnesemia also had a higher incidence of hypercalcemic crisis (47.4% versus 14.1%, *P* < 0.001; Fig. 1). Similarly, both serum PTH and calcium levels were negatively correlated with serum magnesium (*P* < 0.05; Fig. 2). In females, low serum albumin levels were associated with hypomagnesemia. Similarly, there was a decreasing trend in serum albumin levels in males with hypomagnesemia, but it did not reach statistical significance. The femoral neck BMD was significantly lower in the hypomagnesemia group in males (Table 1). Moreover, a weak but significant positive correlation was observed between total hip and femoral neck BMD and serum magnesium (*P* < 0.05; Fig. 2). The hypomagnesemia group had, on average, a lower lumbar and hip T score (Table 2) and a higher prevalence of osteoporosis than the normal magnesium group (58.4% versus 41.7%, *P* < 0.05; Fig. 1).

**Table 1:** Demographic characteristics and serum parameters of study cohort

	Males			Females		
	normal magnesium group(*n* = 69)	hypomagnesemia group(*n* = 33)	P	normal magnesium group(*n* = 161)	hypomagnesemia group(*n* = 44)	P
Age (years)	49.2 ± 16.3	45.6 ± 15.8	0.304	54.9 ± 13.8	52.3 ± 12.0	0.262
BMI (kg/m^2^)	23.0 ± 2.8	20.8 ± 2.9	0.001	22.5 ± 3.5	22.3 ± 4.0	0.772
Hemoglobin (g/L)	134 ± 22	115 ± 23	<0.001	118 ± 18	107 ± 17	<0.001
eGFR (mL/min/1.73 m^2^)	76.2 ± 37.7	65.1 ± 38.5	0.238	80.3 ± 41.0	69.2 ± 34.0	0.141
Creatinine (umol/L)	99.2 ± 60.8	138.0 ± 112.7	0.032	78.6 ± 75.7	91.0 ± 51.7	0.315
Albumin (g/L)	38.6 ± 3.3	37.5 ± 6.5	0.301	39.3 ± 9.4	36.0 ± 6.2	0.026
Serum calcium (mmol/L)	2.81 ± 0.34	3.08 ± 0.46	0.005	2.73 ± 0.37	3.10 ± 0.59	<0.001
Corrected calcium (mmol/L)	3.06 ± 0.45	3.40 ± 0.59	0.004	2.96 ± 0.47	3.50 ± 0.66	<0.001
Serum phosphorus (mmol/L)	0.71 ± 0.20	0.74 ± 0.32	0.823	0.80 ± 0.28	0.80 ± 0.31	0.944
Serum magnesium (mmol/L)	0.94 ± 0.12	0.62 ± 0.12	<0.001	0.93 ± 0.12	0.62 ± 0.12	<0.001
24-h urinary calcium (mmol/day)	7.21 ± 4.26	9.55 ± 8.75	0.250	5.79 ± 3.19	7.51 ± 5.52	0.027
24-h urinary phosphorus (mmol/day)	15.7 ± 5.9	14.3 ± 7.0	0.394	13.0 ± 6.5	10.0 ± 4.6	0.018
24-h urinary magnesium (mmol/day)	2.91 ± 0.94	4.19 ± 3.67	0.046	2.54 ± 1.75	2.87 ± 3.16	0.444
PTH (pg/mL)	46.2 ± 50.1	157.2 ± 116.9	<0.001	50.2 ± 63.5	99.6 ± 90.3	<0.001
25(OH) D (ng/mL)	34.4 ± 18.2	36.6 ± 16.8	0.659	32.4 ± 16.5	34.6 ± 19.2	0.516
Lumbar BMD(g/cm^2^)	0.82 ± 0.16	0.77 ± 0.15	0.172	0.72 ± 0.15	0.69 ± 0.13	0.304
Hip BMD(g/cm^2^)	0.73 ± 0.16	0.64 ± 0.17	0.054	0.65 ± 0.17	0.62 ± 0.15	0.430
FN BMD(g/cm^2^)	0.60 ± 0.14	0.52 ± 0.14	0.024	0.53 ± 0.14	0.51 ± 0.12	0.428

*BMI* body mass index, *eGFR* estimate glomerular filtration rate, *PTH* parathyroid hormone, *25 (OH) D*, 25-hydroxyvitamin D, *BMD* bone mineral density, *FN* femoral neck

**Table 2:** BMD of patients with PHPT

	normal magnesium group(*n* = 136)	hypomagnesemia group(*n* = 52)	P
Lumbar T score	−2.17 ± 1.38	−2.64 ± 1.21	0.032
Lumbar Z score	−0.89 ± 1.46	−1.87 ± 1.40	<0.001
FN T score	−2.67 ± 1.26	−3.07 ± 1.22	0.053
FN Z score	−1.16 ± 1.59	−2.22 ± 1.54	<0.001
Hip T score	−2.04 ± 1.40	−2.49 ± 1.36	0.045
Hip Z score	−0.97 ± 1.47	−1.85 ± 1.63	<0.001

The typical symptoms of PHPT, such as nephrolithiasis, bone pain/fractures, polyuria or polydipsia, were more common in the hypomagnesemia group (Table 3). There was a trend toward more gastrointestinal involvement in the hypomagnesemia group, but it did not reach statistical significance. The incidence of fatigue did not differ between the two groups.

**Table 3:** Clinical presentation of patients with PHPT

	normal magnesium group(*n* = 220)	hypomagnesemia group(*n* = 78)	P
Symptomatic n(%)	157(71.4)	71(91.0)	<0.001
Nephrolithiasis n(%)	104(47.3)	51(65.4)	0.006
Bone pain/fractures n(%)	54(24.5)	31(39.7)	0.011
Polyuria/polydipsia n(%)	28(12.7)	19(24.4)	0.015
Gastrointestinal involvement n(%)	17(7.7)	11(14.1)	0.097
Asymptomatic n(%)	63(28.6)	7(9.0)	<0.001

Overall, the odds ratio (OR) for hypercalcemic crisis in patients with PHPT with hypomagnesemia versus normal magnesium was 5.50 (95% confidence intervals [CI] 3.07–9.87, *P* < 0.001; Table 4). The higher risk of hypercalcemic crisis in the hypomagnesemia group persisted after adjusting for age, sex, BMI, eGFR, and PTH (OR 3.43, 95% CI 1.60–7.33, *P* = 0.001). The OR for anemia in the hypomagnesemia group was 3.30 (*P* < 0.001) and 3.79 (*P* = 0.001) in unadjusted and multivariate adjusted analyses. Hypercalcemic patients also showed a 2.67-fold higher risk for osteoporosis after multivariate adjustment (*P* = 0.006).

**Table 4:** Complications of patients with PHPT

	Unadjusted			Adjusted for age, gender, BMI, eGFR and PTH		
	OR	95% CI	P	OR	95% CI	P
Osteoporosis	1.76	1.05–2.97	0.034	2.67	1.33–5.37	0.006
Anemia	3.30	1.86–5.87	<0.001	3.79	1.76–8.14	0.001
Hypercalcemic Crisis	5.50	3.07–9.87	<0.001	3.43	1.60–7.33	0.001

## Discussion

The prevalence of hypomagnesemia was 25.1% in the total study population, 32.4% in males, and 21.5% in females. The occurrence of hypomagnesemia in PHPT has been noted previously and was confirmed in this study; a significant negative correlation was found between serum calcium and magnesium in patients with PHPT. These results are consistent with those of a previous study that included 73 hospitalized patients with PHPT [18]. Our results indicate that the higher prevalence of hypercalcemic crisis persisted after adjusting for eGFR and PTH levels in patients with hypomagnesemia. Magnesium levels are maintained within a normal range by a dynamic interplay among intestinal absorption, exchange with bone, and renal excretion, and disruption of these processes may cause hypomagnesemia [26, 27]. Magnesium is chiefly eliminated through renal excretion; thus, increased renal excretion leads to hypomagnesemia [28]. Although PTH stimulates an increase in tubular magnesium reabsorption in patients with PHPT, hypercalcemia has the opposite effect [15]. Hypercalcemia can cause hypomagnesemia owing to increased filtered calcium load in the loop of Henle, resulting in decreased reabsorption of magnesium [29]. In the present study, the mean 24 h urinary magnesium levels in the hypomagnesemia group were higher than those in the normal magnesium group in both males and females, but the difference was not statistically significant. This finding may be explained by the fact that we did not measure urinary creatinine and were unable to calculate the renal fractional excretion of magnesium. The renal fractional excretion of magnesium is more effective in evaluating renal magnesium excretion [30].

Hypomagnesemia can also be secondary to impaired intestinal magnesium absorption. A trend of a higher risk of gastrointestinal involvement in the hypomagnesemia group was observed in this study. Moreover, proton pump inhibitors, commonly used in gastrointestinal disorders, have been associated with hypomagnesemia in patients [31, 32]. Our study is limited in this aspect because data on the use of proton pump inhibitors were not obtained.

Our results suggest that the typical symptoms of PHPT were more common in the hypomagnesemia group. Patients with hypomagnesemia had a higher prevalence of nephrolithiasis than patients with normal magnesium, which is consistent with the findings of other studies in the general population [33, 34]. This may be because magnesium—one of the inhibitors of stone formation—competes with calcium to bind to oxalic acid to form magnesium oxalate, a complex that is more easily soluble in urine [35]; at low magnesium levels, this competition is diminished. Hypercalciuria is a well-established risk factor for nephrolithiasis in patients with and without PHPT [36]. The mean 24 h urinary calcium levels in the hypomagnesemia group were higher than those in the normal magnesium group in both males and females, but the difference was not statistically significant.

The incidence of bone pain/fractures and osteoporosis was higher in the hypomagnesemia group than in the normal magnesium group. Even after adjusting for potential confounders such as age, sex, BMI, eGFR, and PTH, the association remained essentially unchanged. This result is consistent with the known effect of hypomagnesemia on the prevalence of osteoporosis in the general population [37–39]. This is likely because low magnesium can alter trabecular bones owing to the formation of large but fragile crystals [40]. Moreover, low magnesium can reduce the vascular supply of bones [41] and increase inflammatory cytokines [42], which promote bone pain and fractures. High PTH activates osteoclasts more readily by enhancing RANKL expression, which increases calcium resorption and bone loss, promoting an osteoporotic state [43]. High calcium can cause renal tubular damage—which decreases the renal tubular concentration function—and increases urinary calcium excretion, which leads to polydipsia and polyuria [44]. This phenomenon may explain the finding that polyuria was more common in the hypomagnesemia group in our study.

The hypomagnesemia group showed significantly lower average hemoglobin levels than the normal magnesium group in both males and females, and the greater prevalence of anemia in the hypomagnesemia group persisted after controlling for the presence of eGFR and PTH. These results suggest that the effect of hypomagnesemia on anemia in PHPT is independent of the biochemical severity of the disease. The higher prevalence of anemia with hypomagnesemia has been described in individuals without PHPT [32, 45–47].

The present study has certain limitations. First, data on other risk factors for hypomagnesemia were not obtained for our patient population, such as the use of proton pump inhibitors. Second, urinary creatinine and ionized and intracellular free calcium and magnesium levels were not assessed, and effects of magnesium supplementation was not studied. Third, BMD data were available only for approximately three-fifth of the cohort, and distal forearm BMD was not assessed. Fourth, these finding are related to a hospitalized PHPT populations. Thus, these relationships cannot be immediately translated to PHPT outpatients. Lastly, considering the retrospective nature of this study, causal inferences between hypomagnesemia and different clinical features and complications of PHPT cannot be assumed.

Despite these limitations, this study had several strengths. First, this study evaluated data from a large cohort of PHPT patients. Second, blood measurements were performed at the same hospital laboratory. Third, we assessed the prevalence of hypomagnesemia in patients with PHPT. Fourth, this is the first study that assessed whether there is any association between serum magnesium and severity of primary hyperparathyroidism. Finally, we found that hypomagnesemia, which is a frequent electrolyte disorder in PHPT patients, is associated with severity of primary hyperparathyroidism. It is not known whether correcting hypomagnesemia with magnesium supplements in PHPT patients will reduce the progression of PHPT and other associated comorbidities. Further prospective studies with a higher number of patients are needed for this purpose.

In conclusion, we found that hypomagnesemia was associated with higher serum calcium and PTH, and clinical symptoms were more common in patients with hypomagnesemia. In addition, PHPT patients with hypomagnesemia had a higher prevalence of osteoporosis, anemia, and hypercalcemic crisis.

## Data Availability

The datasets used and/or analysed during the current study are available from the corresponding author on reasonable request.
